# Indicators of resource scarcity differentially moderate the impact of threat exposure on psychopathology in a cross-sectional community sample of youth

**DOI:** 10.3389/frcha.2025.1568829

**Published:** 2025-09-15

**Authors:** Eric R. Larson, Alexandra B. Moussa-Tooks, Krista M. Wisner

**Affiliations:** ^1^ Psychological and Brain Sciences, Indiana University, Bloomington, IN, United States; ^2^ Program in Neuroscience, Indiana University, Bloomington, IN, United States

**Keywords:** early life adversity, adolescence, threat, scarcity, dimensional psychopathology

## Abstract

Threat exposure and resource scarcity increase psychopathology risk throughout childhood and adolescence. However, it remains unclear whether these dimensions of early life adversity interact to impact psychopathology, whether different indicators of resource scarcity perform similarly in such interactions, and whether these relationships are similar between males and females. This analysis used a cross-sectional, multi-informant approach to investigate interactions between threat exposure and different indicators of resource scarcity (achievement-based, financial-based) for three major dimensions of psychopathology. Data are from 236 community-based non-help seeking youth aged 8-17 (M = 11.58, SD = 2.74) enrolled in the census-matched Nathan Kline Institute-Rockland Sample. Linear models were used to estimate interactions between threat exposure, and achievement-based scarcity (caregiver education and occupation) vs. financial-based scarcity (income-to-needs ratio), for major dimensions of psychopathology (internalizing, externalizing, thought disturbance). Linear models showed increasing threat exposure was associated with elevated internalizing and externalizing psychopathology symptoms, but not thought disturbance symptoms, when controlling for resource scarcity indicators. Achievement-based scarcity, but not financial-based scarcity, moderated these relationships, such that the impact of threat exposure on psychopathology depended on the level of caregiver achievement moreso than on the amount of familial financial resources. These patterns were similar in males (*N* = 132) and females (*N* = 104) when examined separately. Caregiver achievement may protect against symptoms of psychopathology in youth exposed to threat, suggesting that policies geared towards increasing education accessibility and job opportunities may have considerable downstream impact for child and adolescent well-being. Future work should explore interactions between adversity dimensions in population-based samples with greater variability in systems-level factors (e.g., neighborhood advantage).

## Introduction

Early life adversity (ELA, e.g., childhood trauma, socioeconomic disadvantage) is a robust risk factor for the development of psychopathology in childhood and adolescence (1, 2). Novel conceptualizations of ELA posit that different adversities can be categorized into dimensions based on the quality of the environmental deviation they reflect (e.g., threat vs. scarcity) (3). While current evidence suggests dimensions of ELA nonspecifically increase risk for psychopathology (4, 5), little research has tested (a) whether distinct but often co-occurring dimensions of ELA (e.g., threat and resource scarcity) interact to influence psychopathology, and (b) whether interactions between ELA dimensions may depend on the indicator of an adversity dimension (e.g., achievement-based scarcity vs. financial-based scarcity). Prior work has utilized specificity or cumulative risk approaches (6, 7), which consider risk to the individual from exposure to single adversity types or the sum of exposure to distinct adversity types, respectively. Instead, dimensional models can be used to categorize adversity types into broader underlying dimensions reflecting their shared quality of environmental deviation (8, 9). For example, adversities that confer risk of acute harm to an individual could be considered along a *threat* dimension (9), whereas other types of adversities reflecting familial social and economic capital could be characterized along a *resource scarcity* dimension (10).

*Threat* is a heterogenous dimension of adversity defined as exposure to events that pose physical or psychological harm to an individual and is among the strongest predictors of psychopathology in childhood and adolescence (9). Threat exposure is often assayed with measures of childhood trauma (11) and violence exposure (12, 13). Different threat exposures (e.g., abuse, illness/death, and violence) largely confer similar degrees of risk for different psychopathologies (14–17), though certain threat exposures (e.g., childhood sexual abuse) may be particularly potent (18) highlighting the utility of considering these experiences within a shared dimension instead of as single types.

*Resource scarcity*, also a heterogenous dimension of adversity, is characterized by experiences involving a potential lack of access to necessary financial and/or organizational resources (10, 19, 20). Of note, resource scarcity is related to, but distinct from, deprivation, which is conceptualized as the absence of expected environmental input on behalf of the caregiver (e.g., neglect, cognitive stimulation) and is theorized to reflect a unique dimension of adversity (21). One widely-used index of resource scarcity is socioeconomic status/disadvantage. Despite variability in indicators (22), lower socioeconomic status is consistently associated with elevated psychopathology (23); however, the degree of risk may depend on the specific indicator of scarcity. For example, the effect of lower socioeconomic status on psychopathology differs when considering caregiver achievement (e.g., education/occupation) vs. familial financial resources (e.g., financial hardship) (23–26). Accordingly, achievement-based and financial-based resource scarcity may reflect aspects of the construct of scarcity that could be uniquely perceived by a developing individual or affect unique domains of caregiving and are often comprised of multiple indicators (e.g., education, occupation, income, family size). Such differences in the perception of scarcity or the effects of scarcity on parenting behaviors may impact different psychopathology dimensions in youth (27).

Critically, threat and resource scarcity are interrelated. For example, greater resource scarcity is associated with elevated rates of exposure to threatening events, including self-reported adverse childhood experiences (28) and county-level child maltreatment reports (29). Moreover, adverse childhood experiences have been shown to mediate the relationship between resource scarcity and psychopathology in adolescence (30). However, it remains unclear whether resource scarcity moderates the impact of threat on psychopathology. Previous research has shown that caregiver education, but not occupation or family income, moderated the relationship between threat exposure and total psychopathology (31), with caregiver education buffering the negative effect of threat exposure on psychopathology. Others do not detect moderation effects for any indicator of resource scarcity for different dimensions of psychopathology (e.g., internalizing, externalizing) (32), perhaps due to discrepant modelling of ELA dimensions. Accordingly, threat and resource scarcity are related constructs, but reflect distinct environmental perturbances. Thus, their relationship warrants further examination, especially to develop improved risk assessments based on different forms of early life adversity.

Furthermore, the lack of focus on sex differences in these relationships contributes to conflicting findings. Though biological sex is often a covariate, the (in)significance of a covariate is insufficient for determining whether putative causes of psychopathology are sex-invariant (33). Independent of ELA exposure, males and females differ in psychopathology symptoms and trajectories throughout childhood and adolescence, with males generally showing higher externalizing behaviors (e.g., rule-breaking, aggression) and females reporting higher internalizing symptoms (e.g., anxiety, depression, somatic complaints) (34–36). This, combined with evidence that ELA exposure may differ between males and females, including higher rates of sexual abuse (37) and a greater number of data-driven ELA profiles (38) in females than males, suggests that interactions between ELA dimensions could also differ by biological sex. To date, evidence is mixed regarding the direction of sex differences for psychopathology presentations in ELA exposed youth (2, 26, 39–41). Most studies focus on specific adversities or categorical diagnoses rather than dimensional constructs. As such, it remains unclear whether adversity dimensions interact differently in males and females to impact psychopathology dimensions, which is an important consideration when building etiological models of adversity and psychopathology (42).

The current study expands on this existing research by testing three hypotheses. First, we hypothesized that threat exposure is positively related to adolescent psychopathology, including internalizing, externalizing, and thought disturbance symptoms after controlling for environmental scarcity. Second, we hypothesized that each indicator of resource scarcity moderates these effects, such that higher achievement-based scarcity and financial-based scarcity (i.e., lower resource availability) confers greater risk for the adverse impact of threat exposure on adolescent psychopathology. Third, we hypothesized that these moderation effects differ in magnitude when modeled in each sex separately. Given inconsistencies in the literature, we did not specify directional hypotheses regarding potential differences as a function of biological sex.

## Materials and methods

### Participants

Cross-sectional data were obtained from 236 community-based non-help seeking youth aged 8-17 enrolled in the Census-matched Nathan Kline Institute Rockland Sample (NKI-RS) who completed all demographic surveys and self-reported questionnaires (described below). This publicly available dataset is representative of the United States demographics per the 2010 US Census. All data was collected in Rockland County, New York, with full details published elsewhere (43). All in-person study procedures were approved by the Institutional Review Board of the Nathan Kline Institute; Institutional Review Board approval was obtained from Indiana University to download the dataset and conduct secondary data analyses. Full inclusion and exclusion criteria are presented elsewhere (44), but generally encompass prenatal/birth difficulties, neurological impairments, history of substance use disorder and/or psychotropic medication use, and family history of severe psychopathology (e.g., psychotic episode, psychiatric hospitalization, current suicidal ideation).

### Measures

#### Threat exposure

The self-report UCLA Posttraumatic Stress Disorder Reaction Index (45) measures threat exposure for 13 different potentially traumatic events and associated PTSD symptoms (e.g., “Being in a bad accident, like a very serious car accident”, “Seeing a family member being hit, punched, or kicked very hard at home”, “Hearing about the violent death or serious injury of a loved one”). Items related to environmental scarcity are not included in the scale. To create a threat exposure score, the first 13 items were used in which individuals indicate “Yes” or “No” to experiencing a given traumatic event. Caregivers and youth independently completed the exposure measure, and an “either-or” multi-informant approach was used, where exposure to a traumatic experience was considered present if either the caregiver or the youth responded affirmatively to the youth being exposed, consistent with other studies of youth threat exposure (46). Low endorsement of more than three items (i.e., few participants endorsing more than three potentially traumatic events) items warranted creation of a composite score capped at “three or more”; thus, for analyses the range was zero to three.

#### Resource scarcity

Two distinct caregiver-reported indicators of resource scarcity were used in the current study: achievement-based scarcity and financial-based scarcity. *Achievement-based scarcity* was measured with the widely-used Hollingshead Four-Factor Index (47). Scores on the achievement-based scarcity metric were determined through the scale's established and widely-used composite of caregivers' education [Likert scale ranging from 1 (7th Grade) to 7 (Graduate Degree], occupation [Likert scale ranging from 1 (Farm Laborers) to 9 (Executives/Business Owners)], marital status, and employment status. Total scores reflect the sum of caregivers' education score weighted by three, and their occupation score weighted by five; thus, this measure ranged from 8–66. Higher scores represent lower achievement-based scarcity (i.e., higher composite caregiver education and occupation). *Financial-based scarcity* was measured with the family income-to-needs ratio (INR). INR was computed through dividing the median value of the household income band reported by caregivers by the United States federal poverty level for the respective household size. The household income bands in NKI-RS included, in United States Dollars, <10,000, 10,000–14,900, 15,000–24,900, 25,000–34,9000, 35,000–49,900, 50,000–74,900, 75,000–99,900, 100,000–199,900, >200,000. An INR below one is considered below federal poverty level. Higher INR represents lower financial-based scarcity (i.e., higher family financial resources).

#### Dimensional psychopathology

The caregiver-reported Child Behavior Checklist (CBCL) (48) assays internalizing (32 items, e.g., “Nervous, high-strung, or tense”, “Withdrawn, doesn't get involved with others”), externalizing (27 items, e.g., “Can't concentrate, can't pay attention for long”, “Breaks rules at home, school, or elsewhere”), and thought disturbance (15 items, e.g., “Sees things that aren't there”, “Repeats certain acts over and over”) dimensions of psychopathology. Items are rated on a three-point Likert scale (0 = not true, 1 = somewhat or sometimes true, 2 = very often or often true). The broadband externalizing scale subsumes rule-breaking behavior and aggressive behavior subscales. The broadband internalizing scale is comprised of anxious/depressed, withdrawn/depressed, and somatic complain substances. The current study used raw CBCL scores in all analyses given the interest in differences between males and females. Previous research has demonstrated measurement invariance of the CBCL between males and females in community samples (49, 50), enabling meaningful comparisons of scores.

#### Analysis

All analyses were conducted in R (version 4.2.0). To address outliers, variables were Winsorzied into ±3 standard deviations prior to analyses. Differences between males and females in threat exposure, resource scarcity, and psychopathology were assessed using two-tailed t-tests or chi-square tests to contextualize any potential sex differences in relationships or interactions between psychopathology and adversity dimensions. Prior to multivariate linear models, bivariate relationships between threat exposure and i. indicators of resource scarcity and ii. psychopathology were assessed with Spearman correlations; Pearson correlations were used to assess relationships between indicators of resource scarcity and psychopathology dimensions.

Next, a series of linear models were estimated to address primary hypotheses. For all hypotheses, models were tested independently for internalizing, externalizing, and thought disturbance measures as different empirical dimensions of psychopathology of equal *a priori* interest. First (Hypothesis 1), confirmatory additive models estimated the effect of increasing threat exposure on CBCL raw scores while including as covariates age and biological sex, as well as achievement-based and financial-based scarcity. As an exploratory aim, to contextualize the potential specificity of ELA dimensions in comparison to other approaches, the impact of a cumulative risk score on psychopathology was subsequently evaluated while including as covariates age and biological sex. To calculate a cumulative risk score, values in the bottom third of achievement-based scarcity and financial-based scarcity were considered “low” and combined with a binary threat exposure variable indicating exposure to any number of threatening events on the UCLA PTSD Reaction Index. Thus, cumulative risk scores ranged from 0 (no threat exposure and low scarcity on both indicators) to 3 (threat exposure and high scarcity on both indicators). Second (Hypothesis 2), to test for moderation effects, linear models estimated the interaction between (a) threat and achievement-based scarcity, and separately (b) threat and financial-based scarcity, on CBCL raw scores while including as covariates age, biological sex, and the resource scarcity indicator that was not the primary focus. Significant interactions were followed-up with Johnson-Neyman region of significance testing to identify at what level of the moderator (i.e., scarcity indicator) the regression slope (i.e., threat exposure on psychopathology) was significant (51). Comparisons between nested models (interactions in Hypothesis 2 and additive effects in Hypothesis 1) were evaluated with one-way analysis of variance to assess improvements in model fit. Third (Hypothesis 3), to test whether observed relationships or interactions were similar between sexes, the sample was split into males and females and the above interaction models were repeated in each group independently while controlling for age. We elected not to include sex as a third interaction term and instead report interaction in males and females separately given (a) the *a priori* question of interest was in whether general patterns were similar or not between males and females, and not whether the interaction was statistically significantly different between sexes and (b) the sample is not adequately powered to test three-way interactions. Threat and resource scarcity are disproportionately distributed among racial minorities due to systemic racism and biases (52). Accordingly, including race as a covariate may lead to inappropriate interpretation of effects (i.e., if inclusion of race renders adversity dimension effects null). Thus, race was not included as a covariate, consistent with other analyses of ELA data (53, 54).

## Results

### Sample characteristics and bivariate correlations

Descriptive statistics for demographics, threat exposure, resource scarcity, and psychopathology in the full sample (*N* = 236) are presented in Table 1. Included youth ranged from ages 8-17 (M = 11.6, SD = 2.7). Majority of the sample was white (70% white, 17% black, 13% other/mixed race). The average household size was 4.25 members (SD = 1.1) and 52% of the youth had married parents. These household variables were not related to threat exposure or either indicator of environmental scarcity (Supplementary Materials). Males (*n* = 132) and females (*n* = 104) did not differ in any demographic or household variable, threat exposure, metric of environmental scarcity, or dimension of psychopathology. Bivariate correlations between threat exposure, resource scarcity, and psychopathology are presented in Table 2. Of note, threat exposure was not correlated with either indicator of resource scarcity in this sample. Achievement-based and financial-based scarcity were significantly correlated at a moderate level (*r* = 0.48, *p* < 0.001).

**Table 1:** Descriptive statistics for All study variables.

Variable	Full Sample	Males	Females	*χ*2 or *t*	*p*
	Frequency or Mean (SD)	Frequency or Mean (SD)	Frequency or Mean (SD)		
*N*	236	132	104		
Age	11.58 (2.74)	11.59 (2.79)	11.57 (2.70)	0.07	0.95
Race (White/Black/Other)	166/40/30	92/25/15	74/15/15	1.15	0.56
Caregiver Marital Status	160/2/29/16/29	88/1/17/6/20	72/1/12/10/9	4.37	0.36
Household Size (# members)	4.25 (1.1)	4.3 (1.1)	4.2 (1.1)	0.59	0.56
Threat Exposure (0/1/2/3+)	81/66/49/40	44/39/25/24	37/27/24/16	1.01	0.78
Achievement-Based Scarcity	49.01 (8.82)	48.70 (9.09)	49.41 (8.51)	−0.61	0.54
Financial-Based Scarcity	3.22 (1.82)	3.21 (1.72)	3.23 (1.94)	−0.08	0.93
Internalizing CBCL	7.23 (6.69)	7.14 (6.15)	7.35 (7.35)	−0.24	0.81
Externalizing CBCL	6.64 (6.82)	6.90 (6.75)	6.30 (6.93)	0.67	0.50
Thought Disturbance CBCL	1.48 (1.86)	1.55 (1.79)	1.38 (1.96)	0.71	0.48

Chi-Square tests were used to estimate differences between males and females on Threat Exposure. All other comparisons used two-tailed t-tests. Frequency variables are N, Race, Caregiver Martial Status, and Threat Exposure. Race was coded as “White”, “Black”, or “Other.” Marial Status was coded as Married/Widowed/Divorced/Separated/Never Married. Threat exposure was coded as 0 (no exposure to threatening events), 1 (exposure to one threatening event), 2 (exposure to two threatening events) or 3+ (exposure to three or more threatening events) per the UCLA PTSD Reaction Index. All other variables are presented as Mean (SD). CBCL, child behavior checklist.

**Table 2:** Bivariate correlations for key study variables.

Variable	1.	2.	3.	4.	5.	6.
1. Threat Exposure	–					
2. Achievement-Based Scarcity	−0.02	–				
3. Financial-Based Scarcity	−0.05	0.48***	–			
4. Internalizing CBCL	0.17*	−0.10	−0.08	–		
5. Externalizing CBCL	0.17**	−0.13*	−0.09	0.62***	–	
6. Thought Disturbance CBCL	0.09	−0.06	−0.003	0.67***	0.59***	–

Correlations involving threat exposure reflect Spearman's rho coefficients. All other correlations reflect Pearson's r coefficients. CBCL, child behavior checklist.

* *p* < 0.5.

** *p* < 0.01.

*** *p* < 0.001.

### Hypothesis 1: confirmatory additive models of ELA

Threat exposure was significantly positively associated with internalizing [*β* = 1.28, 95% CI (0.50, 2.06), *p* < 0.01], and externalizing [*β* = 0.98, 95% CI (0.19, 1.78), *p* = 0.02] psychopathology, but was not associated with thought disturbance psychopathology [*β* = 0.16, 95% CI (−0.05, 0.39), *p* = 0.13] (Table 3). Neither achievement-based scarcity nor financial-based scarcity independently predicted internalizing, externalizing, or thought disturbance dimensions of psychopathology. Notably, this specificity of ELA dimensions on psychopathology was masked by cumulative risk scores of ELA, which were significantly positively associated with internalizing [*β* = 1.22, 95% CI (0.27, 2.15), *p* = 0.01] and externalizing [*β* = 1.15, 95% CI (0.21, 2.10), *p* = 0.02] psychopathology, but not thought disturbance psychopathology [*β* = 0.17, 95% CI (−0.09, 0.43), *p* = 0.22], providing further evidence that simple aggregate scores may obscure unique developmental impacts of different dimensions of environmental deviations.

**Table 3:** Linear models between threat exposure and psychopathology.

Variable	β	*t*	95% CI LL	95% CI UL	*p*
*Internalizing CBCL*	*F*(5, 230) = 2.71; *p* = 0.02; adjusted R^2^ = 0.04				
Age	0.06	0.38	−0.25	0.37	0.70
Biological Sex	0.31	0.36	−1.38	2.01	0.72
Achievement-Based Scarcity	−0.06	−1.17	−0.17	0.04	0.24
Financial-Based Scarcity	−0.11	−0.39	−0.64	0.43	0.69
**Threat Exposure**	1.28	3.24	0.50	2.07	0.001
*Externalizing CBCL*	*F*(5, 230) = 3.24; *p* = 0.008; adjusted R^2^ = 0.05				
Age	−0.33	−2.04	−0.64	−0.01	0.042
Biological Sex	−0.51	−0.58	−2.23	1.21	0.56
Achievement-Based Scarcity	−0.10	−1.70	−0.21	0.02	0.09
Financial-Based Scarcity	−0.02	−0.08	−0.56	0.52	0.94
**Threat Exposure**	0.99	2.46	0.20	1.78	0.015
*Thought Disturbance CBCL*	*F*(5, 230) = 0.91; *p* = 0.48; adjusted R^2^ = −0.002				
Age	−0.03	−0.66	−0.12	0.06	0.51
Biological Sex	−0.16	−0.65	−0.64	0.32	0.52
Achievement-Based Scarcity	−0.02	−1.09	−0.05	0.01	0.28
Financial-Based Scarcity	0.05	0.70	−0.10	0.21	0.49
**Threat Exposure**	0.17	1.51	−0.05	0.39	0.13

Biological sex was coded as 0 = Male, 1 = Female. CBCL, child behavior checklist. Bold text indicates independent variable of interest. LL, lower limit; UL, upper limit.

### Hypothesis 2: moderation models to test ELA interactions

**Figure 1:**
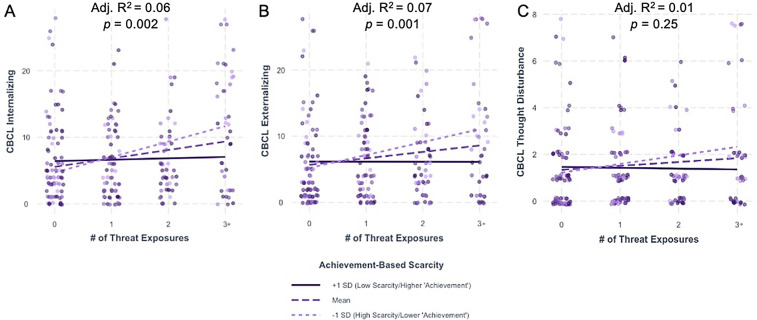
Achievement-based scarcity significantly moderates the impact of threat exposure on internalizing and externalizing psychopathology but not thought disturbance psychopathology. Interactions between threat exposure and achievement-based scarcity on **(A)** internalizing, **(B)** externalizing, and **(C)** thought disturbance CBCL raw scores. Adjusted R^2^ and *p*-value represent those for the overall model. For visualization purposes only, data are split into one standard deviation above (solid dark line) and below (light dotted line) the mean (dashed line). CBCL, child behavior checklist; SD, standard deviation.

Significant interactions were observed between threat exposure and achievement-based scarcity on internalizing [Figure 1A; *β* = −0.12, 95% CI (−0.21, −0.03), *p* < 0.01] and externalizing [Figure 1B; *β* = −0.11, 95% CI (−0.20, −0.02), *p* = 0.01] psychopathology, with no significant interaction on thought disturbance psychopathology [Figure 1C; *β* = −0.02, 95% CI (−0.05, 0.001), *p* = 0.07] (Table 4). That is, youth exposed to increasing amounts of threat exhibited elevated psychopathology only when caregiver composite education and occupation scores were lower. Johnson-Neyman region of significance testing indicated that the linear relationship between threat exposure and psychopathology was significant when achievement-based scarcity was below 52.18 and 49.97 (out of 66) for internalizing and externalizing psychopathology, respectively (Supplementary Figure S1). Furthermore, including achievement-based scarcity as a moderator provided significantly better model fit to the data compared to corresponding additive models for internalizing [*F*(1, 229) = 7.53, *p* < 0.01] and externalizing psychopathology [*F*(1, 229) = 6.27, *p* = 0.01], but not thought disturbance psychopathology [*F*(1, 229) = 3.36, *p* = 0.07].

**Table 4:** Interaction models between threat exposure and achievement-based scarcity.

Variable	β	*t*	95% CI LL	95% CI UL	*p*
*Internalizing CBCL*	*F*(6, 229) = 3.58, *p* = 0.002, adjusted R^2^ = 0.06				
Age	0.08	0.52	−0.23	0.39	0.60
Biological Sex	0.27	0.33	−1.40	1.95	0.75
Financial-Based Scarcity	−0.09	−0.34	−0.62	0.44	0.74
Achievement-Based Scarcity	0.10	1.20	−0.06	0.26	0.23
Threat Exposure	7.12	3.29	2.86	11.38	0.001
Achievement-Based Scarcity * Threat Exposure	−0.12	0.04	−0.21	−0.03	0.007
*Externalizing CBCL*	*F*(6, 229) = 3.81, *p* = 0.001, adjusted R^2^ = 0.07				
Age	−0.31	1.94	−0.62	0.01	0.05
Biological Sex	−0.54	−0.62	−2.24	1.16	0.53
Financial-Based Scarcity	−0.01	−0.03	−0.54	0.53	0.98
Achievement-Based Scarcity	0.05	0.66	−0.11	0.22	0.51
Threat Exposure	6.40	2.91	2.07	10.74	0.004
Achievement-Based Scarcity * Threat Exposure	−0.11	−2.51	−0.20	−0.02	0.013
*Thought Disturbance CBCL*	*F*(6, 229) = 1.33, *p* = 0.25, adjusted R^2^ = 0.01				
Age	−0.03	−0.58	−0.11	0.06	0.57
Biological Sex	−0.16	−0.68	−0.64	0.32	0.50
Financial-Based Scarcity	0.06	0.74	−0.09	0.21	0.49
Achievement-Based Scarcity	0.01	0.59	−0.03	0.06	0.55
Threat Exposure	1.29	2.07	0.07	2.50	0.039
Achievement-Based Scarcity * Threat Exposure	−0.02	−1.83	−0.05	0.00	0.068

Biological sex was coded as 0 = Male, 1 = Female. CBCL, child behavior checklist. Bold text indicates independent variable of interest. LL, lower limit; UL, upper limit.

**Figure 2:**
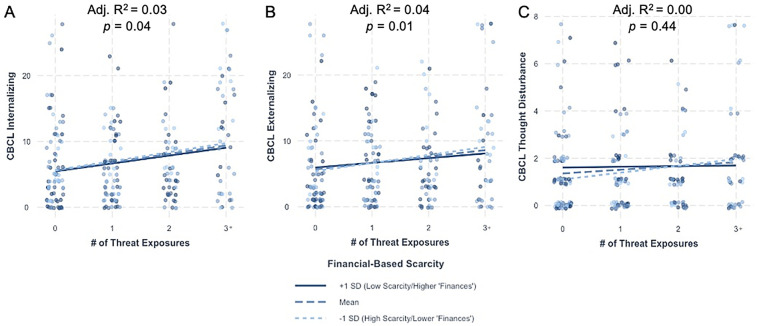
Financial-based scarcity does not moderate the impact of threat exposure on internalizing, externalizing, or thought disturbance psychopathology. Interactions between threat exposure and financial-based scarcity on **(A)** internalizing, **(B)** externalizing, and **(C)** thought disturbance CBCL raw scores. Adjusted R^2^ and *p*-value represent those for the overall model. For visualization purposes only, data are split into one standard deviation above (solid dark line) and below (light dotted line) the mean (dashed line). CBCL, child behavior checklist; SD, standard deviation.

There were no significant interactions between threat exposure and financial-based scarcity on any dimension of psychopathology [Figures 2A–C; Internalizing: *β* = −0.05, 95% CI [−0.48, 0.39], *p* = 0.83; Externalizing: *β* = −0.14, 95% CI [−0.57, 0.30], *p* = 0.53; Thought Disturbance: *β* = −0.07, 95% CI [−0.19, 0.05], *p* = 0.25] (Supplementary Table S1). Models with financial-based scarcity as a moderator did not improve model fit compared to corresponding additive models [Internalizing: F(1, 229) = 0.04, *p* = 0.83; Externalizing: F(1, 229) = 0.09, *p* = 0.77; Thought Disturbance: F(1, 229) = 1.33, *p* = 0.25].

### Hypothesis 3: exploration of sex differences in ELA interactions

**Figure 3:**
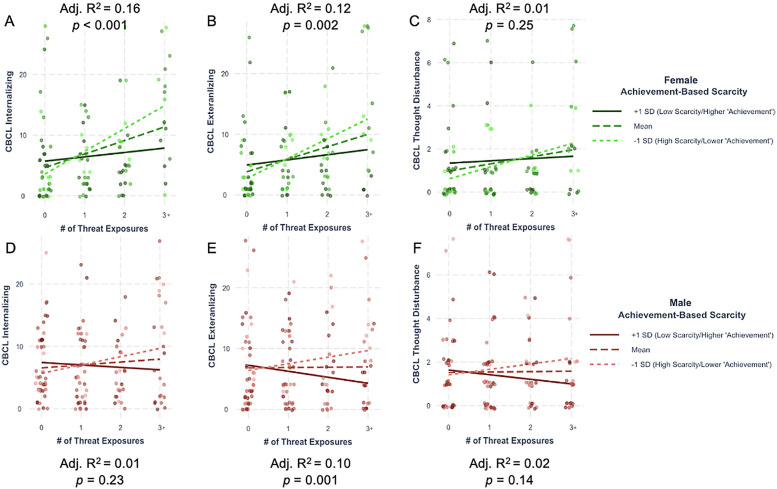
Interactions between threat exposure and achievement-based scarcity on internalizing, externalizing, and thought CBCL raw scores in each biological sex. Females shown in panels **(A-C)** (internalizing, externalizing, thought). Males shown in panels **(D-F)** (internalizing, externalizing, thought). Adjusted R^2^ and *p*-value represent those for the overall model. For visualization purposes only, data are split into one standard deviation above (solid dark line) and below (light dotted line) the mean (dashed line). CBCL, child behavior checklist; SD, standard deviation.

First, there was a significant interaction between threat exposure and achievement-based scarcity for internalizing psychopathology in females [*β* = −0.17, 95% CI (−0.40, −0.03), *p* = 0.01] and a trend-level effect in males [*β* = −0.09, 95% CI (−0.20, 0.01), *p* = 0.08], There were also significant interactions between threat exposure and achievement-based scarcity for externalizing psychopathology in both females [*β* = −0.14, 95% CI (−0.27, −0.005), *p* = 0.042] and males [*β* = −0.11, 95% CI (−0.22, −0.003), *p* = 0.044] (Tables 5, 6, Figure 3). These effects are in the same direction as the combined sample. There were no significant or trend-level interactions between threat exposure and achievement-based scarcity for thought disturbance psychopathology in females [*β* = −0.02, 95% CI (−0.06, 0.01), *p* = 0.20] or males [*β* = −0.02, 95% CI (−0.05, 0.01), *p* = 0.10]. Finally, there were no significant or trend-level interactions between threat and financial-based scarcity for any dimension of psychopathology in either sex (Supplementary Tables S2, S3; Supplementary Figure S2).

**Table 5:** Females interaction models between threat exposure and achievement-based scarcity.

Variable	β	*t*	95% CI LL	95% CI UL	*p*
*Internalizing CBCL*	*F*(4, 99) = 6.05, *p* < 0.001, adjusted R^2^ = 0.16				
Age	0.40	1.59	−0.10	0.89	0.11
Achievement-Based Scarcity	0.12	0.96	−0.13	0.38	0.34
Threat Exposure	11.04	3.08	3.93	18.15	0.003
Achievement-Based Scarcity * Threat Exposure	−0.18	−2.50	−0.32	−0.04	0.014
*Externalizing CBCL*	*F*(4, 99) = 4.37, *p* = 0.002, adjusted R^2^ = 0.12				
Age	0.15	0.62	−0.33	0.63	0.54
Achievement-Based Scarcity	0.13	1.04	−0.12	0.38	0.30
Threat Exposure	9.09	2.62	2.19	15.98	0.010
Achievement-Based Scarcity * Threat Exposure	−0.14	−2.06	−0.28	−0.01	0.042
*Thought Disturbance CBCL*	*F*(4, 99) = 1.4, *p* = 0.25, adjusted R^2^ = 0.01				
Age	0.07	0.93	−0.08	0.21	0.36
Achievement-Based Scarcity	0.04	1.14	−0.03	0.12	0.26
Threat Exposure	1.62	1.57	−0.43	3.68	0.12
Achievement-Based Scarcity * Threat Exposure	−0.03	−1.27	−0.07	0.01	0.20

CBCL, child behavior checklist. Bold text indicates independent variable of interest. LL, lower limit; UL, upper limit. Because of power limitations in the split samples, the other indicator of environmental scarcity was not included as a covariate.

**Table 6:** Males interaction models between threat exposure and achievement-based scarcity.

Variable	β	*t*	95% CI LL	95% CI UL	*p*
*Internalizing CBCL*	*F*(4, 127) = 1.43, *p* = 0.23, adjusted R^2^ = 0.01				
Age	−0.18	−0.95	−0.57	0.20	0.34
Achievement-Based Scarcity	0.10	1.04	−0.09	0.28	0.30
Threat Exposure	5.08	1.93	−0.12	10.28	0.056
Achievement-Based Scarcity * Threat Exposure	−0.09	0.05	−0.20	0.01	0.08
*Externalizing CBCL*	*F*(4, 127) = 4.58, *p* = 0.001, adjusted R^2^ = 0.10				
Age	−0.67	−3.29	−1.07	−0.27	0.001
Achievement-Based Scarcity	0.05	0.48	−0.14	0.24	0.63
Threat Exposure	5.60	2.03	0.14	11.06	0.044
Achievement-Based Scarcity * Threat Exposure	−0.11	−2.03	−0.22	−0.00	0.044
*Thought Disturbance CBCL*	*F*(4, 127) = 1.75, *p* = 0.14, adjusted R^2^ = 0.02				
Age	−0.09	−1.59	−0.20	0.02	0.11
Achievement-Based Scarcity	0.01	0.45	−0.04	0.06	0.65
Threat Exposure	1.25	1.65	−0.25	2.76	0.10
Achievement-Based Scarcity * Threat Exposure	−0.03	−1.64	−0.06	0.01	0.10

## Discussion

Different dimensions of early life adversity (ELA), including threat exposure and resource scarcity, are risk factors for psychopathology throughout development. However, studies testing whether dimensions of adversity interact in their impact on dimensional psychopathology, or if effects differ as a function of biological sex, are scarce. These are important considerations when building etiological models of adversity and psychopathology, and for developing appropriate prevention and intervention programs.

The dimensional framework used in the current study strikes a middle-ground between the widely used, but theoretically limited, specificity approach (i.e., examining one type of ELA) and cumulative risk approach (e.g., summed exposure to a range of different binarized ELAs) to conceptualizing ELA. In the present work, threat exposure, operationalized as exposure to a host of different traumatic events, was associated with internalizing and externalizing psychopathology to a similar degree even when controlling for two indicators of environmental scarcity, providing further support that exposure to threatening events is a transdiagnostic risk factor for psychopathology early in development (11). This relationship was expected, given threat exposure is a robust predictor of psychopathology in children (2), adolescents (1), and adults (55). However, contrary to predictions, there was not a significant relationship between threat exposure and the thought disturbance dimension of psychopathology. The thought disturbance CBCL subscale combines various experiences, including items about sleep problems, obsessions/tics, and psychotic-like experiences. While previous work has documented relationships between threat exposure and each of these more specific symptom sets (17, 56, 57), the non-specificity of this CBCL subscale combined with low variability in this sample may have obscured the relationship for thought disturbance psychopathology.

Resource scarcity, operationalized in two forms as either achievement-based scarcity (*via* a composite of caregiver education and occupation) or financial-based scarcity (*via* the income-to-needs ratio), did not evidence any linear relationships with psychopathology in multivariate models (additive or interactive). No associations between achievement-based nor financial-based scarcity with psychopathology emerged in our additive model. A previous meta-analysis has shown different indicators of environmental scarcity, such as caregiver education, familial income, and receipt of public assistance are associated with both internalizing and externalizing psychopathology in adolescence (23). One reason these broader effects may not have been replicated in the current study is because participants in the Nathan Kline Institute—Rockland Sample are recruited from the same geographical area and thus reside in areas with comparable systems-level advantages and disadvantages (43), which limits our range. Structural and organizational resources in the area, such as the availability of housing, insurance coverage and quality, and public infrastructure, may buffer against family-level disadvantages. Future work in wide-ranging population-based samples is needed to understand the role of broader socioecological levels in the impact of family-level ELA on psychopathology in adolescence.

The primary aim of this investigation was to elucidate how different dimensions of ELA interact to affect psychopathology in youth. The interplay between threat exposure and resource scarcity is complex, with evidence suggesting that environmental scarcity is a risk factor for threat exposure itself (28, 29) and that threat exposure is a mediator between environmental scarcity and psychopathology (30, 58). However, the question of moderation, or “for whom” threat exposure is most consequential via the unique interactions of variables of interest, has remained unclear given conflicting previous reports (31, 32). In the current investigation, the critical finding was that achievement-based and financial-based indicators of resource scarcity differentially impacted the relationship between threat exposure and psychopathology in youth, conceptually replicating Reiss et al. (2019). Achievement-based scarcity moderated the relationship between threat exposure and internalizing and externalizing psychopathology. That is, lower achievement-based scarcity (i.e., higher composite caregiver education and occupation) buffered against the negative impact of increasing exposure to threat for internalizing and externalizing behaviors, whereas higher achievement-based scarcity (i.e., lower composite caregiver education and occupation) conferred greater risk for psychopathology in threat-exposed youth. In contrast, there were no moderation effects of financial-based scarcity on threat exposure's relationship with any dimension of psychopathology. That is, no advantage or disadvantage of higher or lower financial-based scarcity (i.e., higher or lower income-to-needs) for psychopathology was observed in this sample from Rockland County, New York. However, our results suggest that caregivers with higher education and occupation may also have greater access to financial resources. Thus, while we did not observe effects of financial resources alone on dimensions of psychopathology, it is possible that higher-achieving caregivers have a source of financial resources that could be leveraged in times of need. Future research testing interactions between indicators of resource scarcity will be invaluable in parsing synergistic or buffering effects of this dimension of ELA on psychopathology. Overall, our findings are consistent with research suggesting that different dimensions of resource scarcity reflect different experiences (perhaps via availability to different types of resources) for families, which appear to confer risk for youth psychopathology, possibly through mechanisms such as impacts on caregiver behaviors (27, 59). Additionally, though our observed effect sizes are relatively modest, they are consistent with research suggesting estimates of effect sizes from community and/or population-based samples are typically smaller than traditional heuristics cutoffs, as well as consistent with effect sizes observed in the broader individual-differences literature (60, 61).

Biological sex is an important sociodemographic consideration in research on ELA and psychopathology (42, 62), but is not often systematically investigated. The present study found the associations between threat exposure and psychopathology were in the same direction across sexes, though larger in females compared to males, consistent with previous work (39, 41, 63). However, the current study did not test for statistical difference in magnitude between sexes due to limited power for three-way interactions of subtle effects. Importantly, the pattern of achievement-based scarcity, but not financial-based scarcity, moderating the relationship between threat exposure and psychopathology was present in both males and females. However, when tested independently in each sex, effects were attenuated, likely reflecting the lower statistical power. These findings cannot be attributed to different levels of threat exposure, resource scarcity, or psychopathology between sexes. Understanding the role of biological sex in ELA impact, even if differences are non-significant, is an important future consideration in developing etiological models of psychopathology (42). Though the NKI-RS dataset does not assess pubertal status, individual differences within biological sex in pubertal development may provide more granular insight into the role of biological sex differences in ELA-associated psychopathology in future studies. Additionally, the time at which threat exposures occurred could interact with sex-specific trajectories in dimensional psychopathology and neurodevelopment (64, 65). Thus, future research is needed to further clarify sex differences.

This study is subject to several limitations that should be addressed in future research. First, the current study used a cross-sectional sample of youth and thus findings do not indicate causality, temporal directionality, or persistence of effects. Second, as noted above, participants were from the same geographical area. While demographically representative of the United States population, most participants presumably had similar systems-level advantages (e.g., housing availability, school quality) that may serve as risk or protective factors above-and-beyond family-level indicators of scarcity. Future research should examine whether the current moderation effects are replicated when including participants from geographical regions with greater variability in these systems-level indicators of resource scarcity. Third, though evidence suggests that subjective indicators of scarcity (e.g., rating one's means relative to their peers or community) are more strongly related to psychopathology than objective measures like caregiver education and income-to-needs (66, 67), the NKI dataset does not have information on subjective impressions of scarcity. Fourth, because of missing data, we were unable to meaningfully examine youth-reported psychopathology symptoms and relied on caregiver's report of their child/adolescent's behavior. Often, caregiver and youth reports of psychopathology differ (68). Thus, it will be important to compare whether threat exposure, achievement-based scarcity, and financial-based scarcity show similar patterns when considering both caregiver and youth-reported psychopathology within a single sample. Fifth, consistent with existing literature, age was a significant predictor of externalizing symptoms, with younger youth demonstrating elevated symptoms. While this may reflect our sample including more males than females, this may also be a function of the development of executive function occurring around this time, which is known to correlate with reduced externalizing symptoms (69). Future work will benefit from testing specifically how age interacts with ELA dimensions to influence psychopathology. Sixth, the current study used a top-down approach to conceptualize threat and resource scarcity (i.e., the research team defined childhood trauma as threat exposure and caregiver education, occupation, and income-to-needs as different indicators of environmental scarcity). Recent advancements in modeling ELA suggest data-driven or person-centered approaches to conceptualizing ELA impact may better reflect “reality” in how the dimensions of ELA form and relate to one another (70, 71). Future work would benefit from integrating these computational approaches with the question of “for whom” adversity may confer greatest risk.

## Conclusion

This study examined whether different dimensions of early life adversity interacted to impact youth psychopathology. Increased threat exposure was associated with elevated internalizing and externalizing, but not thought disturbance, psychopathology symptoms in a community sample of children and adolescents. The impact of threat exposure on psychopathology appeared to be buffered by higher levels of caregiver achievement (i.e., education and occupation), but not financial income-to-needs. Future research should continue to systematically parse the mechanisms by which caregiver education and occupation provide this apparent buffer for youth, as well as identify which systems-level elements (e.g., education, area deprivation) may be important when considering the impact of family-level indicators of environmental scarcity. These data underscore the potential importance of directing policy towards increasing educational opportunities for caregivers in addition to poverty alleviation programs.

## Data Availability

Publicly available datasets were analyzed in this study. Data used in the current study is publicly available via the Nathan Kline Institute pending receipt and approval of a data use agreement (https://fcon_1000.projects.nitrc.org/indi/enhanced/).
