# Supplementary material for: Indicators of resource scarcity differentially moderate the impact of threat exposure on psychopathology in a cross-sectional community sample of youth
Source: Front Child Adolesc Psychiatry. 2025 Sep 15;4:1568829. doi: 10.3389/frcha.2025.1568829 (PMC12477182; doi:10.3389/frcha.2025.1568829)
Supplement: Supplementary file 1 [file Table1.docx]

***Relationships between Household Variables, Threat, and Environmental Scarcity***

There was no difference in threat exposure among martial status groups (*F*(4, 231) = 0.21, *p* = 0.93). There was no relationship between household size and threat exposure (Spearman’s *rho* = -0.08, *p* = 0.21). Similarly, there was no difference in achievement-based scarcity as a function of marital status (*F*(4, 231) = 1.87, *p* = 0.12), though married households had lower financial-based scarcity than divorced (*p* < 0.01) and never married (*p* < 0.05) caregivers (overall ANOVA: (*F*(4, 231)=5.45, *p* < 0.001)). Similarly, there was no relationship between household size and achievement-based scarcity (Spearman’s *rho* = 0.06, *p* = 0.38) or financial-based scarcity (Spearman’s *rho* = -0.04, *p* = 0.53).

| Supplemental Table 1. Interaction Models between Threat Exposure and Financial-Based Scarcity | | | | | | |  |  |
| --- | --- | --- | --- | --- | --- | --- | --- | --- |
|  | β | *t* | | 95% CI LL | 95% CI UL | *p* | | |
| *Internalizing CBCL* | *F*(6, 229) = 2.26, *p* = 0.04, adjusted R^2^ = 0.03 | | | | | |  |  |
| Age | 0.06 | 0.39 | | -0.25 | 0.38 | 0.70 | | |
| Biological Sex | 0.30 | 0.35 | | -1.40 | 2.01 | 0.73 | | |
| Achievement-Based Scarcity | -0.06 | -1.14 | | -0.17 | 0.05 | 0.26 | | |
| Financial-Based Scarcity | -0.05 | -0.14 | | -0.80 | 0.69 | 0.89 | | |
| Threat Exposure | 1.42 | 1.83 | | -0.11 | 2.95 | 0.07 | | |
| **Financial-Based Scarcity * Threat Exposure** | -0.05 | -0.21 | | -0.48 | 0.39 | 0.83 | | |
|  |  |  | |  |  |  | | |
| *Externalizing CBCL* | *F*(6, 229) = 2.76, *p* = 0.013, adjusted R^2^ = 0.04 | | | | | |  |  |
| Age | -0.32 | -2.00 | -0.64 | | -0.01 | 0.046 | |  |
| Biological Sex | -0.53 | -0.61 | -2.26 | | 1.19 | 0.54 | |  |
| Achievement-Based Scarcity | -0.09 | -1.64 | -0.20 | | 0.02 | 0.10 | |  |
| Financial-Based Scarcity | 0.14 | 0.38 | -0.61 | | 0.90 | 0.71 | |  |
| Threat Exposure | 1.41 | 1.80 | -0.14 | | 2.96 | 0.07 | |  |
| **Financial-Based Scarcity * Threat Exposure** | -0.14 | -0.63 | -0.58 | | 0.30 | 0.53 | |  |
|  |  |  |  | |  |  | |  |
| *Thought Disturbance CBCL* | *F*(6, 229) = 0.98, *p* = 0.44, adjusted R^2^ = 0.00 | | | | | |  |  |
| Age | -0.03 | -0.61 | -0.12 | | 0.06 | 0.55 | |  |
| Biological Sex | -0.17 | -0.70 | -0.65 | | 0.31 | 0.48 | |  |
| Achievement-Based Scarcity | -0.02 | -0.99 | -0.05 | | 0.02 | 0.33 | |  |
| Financial-Based Scarcity | 0.14 | 1.31 | -0.07 | | 0.35 | 0.19 | |  |
| Threat Exposure | 0.39 | 1.76 | -0.05 | | 0.82 | 0.08 | |  |
| **Financial-Based Scarcity * Threat Exposure** | -0.07 | -1.15 | -0.19 | | 0.05 | 0.25 | |  |
| Note: Biological sex was coded as 0 = Male, 1 = Female. CBCL = Child Behavior Checklist. Bold text indicates independent variable of interest. LL = lower limit, UL = upper limit. | | | | | | | |  |

| Supplemental Table 2. Females Interaction Models between Threat Exposure and Financial-Based Scarcity | | | | | |  |  |
| --- | --- | --- | --- | --- | --- | --- | --- |
|  | β | *t* | 95% CI LL | 95% CI UL | *p* | | |
| *Internalizing CBCL* | *F(*4, 99) = 4.09, *p* = 0.004, adjusted R^2^ = 0.11 | | | | |  |  |
| Age | 0.37 | 1.45 | -0.14 | 0.88 | 0.15 | | |
| Financial-Based Scarcity | -0.02 | -0.04 | -1.15 | 1.10 | 0.97 | | |
| Threat Exposure | 3.09 | 2.63 | 0.76 | 5.42 | 0.009 | | |
| **Financial-Based Scarcity * Threat Exposure** | -0.29 | 0.32 | -0.92 | 0.34 | 0.36 | | |
|  |  |  |  |  |  | | |
| *Externalizing CBCL* | *F*(4, 99) = 3.75, *p* = 0.01, adjusted R^2^ = 0.10 | | | | |  |  |
| Age | 0.11 | 0.45 | -0.37 | 0.59 | 0.66 | |  |
| Financial-Based Scarcity | 0.48 | 0.90 | -0.58 | 1.55 | 0.37 | |  |
| Threat Exposure | 3.56 | 3.20 | 1.35 | 5.77 | 0.002 | |  |
| **Financial-Based Scarcity * Threat Exposure** | -0.49 | -1.63 | -1.09 | 0.11 | 0.11 | |  |
|  |  |  |  |  |  | |  |
| *Thought Disturbance CBCL* | *F*(4, 99) = 1.69, *p* = 0.16, adjusted R^2^ = 0.03 | | | | |  |  |
| Age | 0.05 | 0.70 | -0.09 | 0.19 | 0.49 | |  |
| Financial-Based Scarcity | 0.23 | 1.49 | -0.08 | 0.55 | 0.14 | |  |
| Threat Exposure | 0.79 | 2.44 | 0.15 | 1.44 | 0.017 | |  |
| **Financial-Based Scarcity * Threat Exposure** | -0.15 | -1.70 | -0.33 | 0.03 | 0.09 | |  |
| Note: CBCL = Child Behavior Checklist. Bold text indicates independent variable of interest. LL = lower limit, UL = upper limit. Because of power limitations in the split samples, the other indicator of environmental scarcity was not included as a covariate. | | | | | | |  |

| Supplemental Table 3. Males Interaction Models between Threat Exposure and Financial-Based Scarcity | | | | | | |  |  |
| --- | --- | --- | --- | --- | --- | --- | --- | --- |
|  | β | *t* | | 95% CI LL | 95% CI UL | *p* | | |
| *Internalizing CBCL* | *F*(4, 127) = 0.75, *p* = 0.56, adjusted R^2^ = -0.01 | | | | | |  |  |
| Age | -0.19 | -0.98 | | -0.59 | 0.20 | 0.33 | | |
| Financial-Based Scarcity | -0.34 | -0.74 | | -1.25 | 0.57 | 0.46 | | |
| Threat Exposure | -0.23 | -0.23 | | -2.29 | 1.82 | 0.82 | | |
| **Financial-Based Scarcity * Threat Exposure** | 0.24 | 0.82 | | -0.35 | 0.84 | 0.42 | | |
|  |  |  | |  |  |  | | |
| *Externalizing CBCL* | *F*(4, 127) = 2.97, *p* = 0.02, adjusted R^2^ = 0.06 | | | | | |  |  |
| Age | -0.67 | -3.19 | -1.08 | | -0.25 | 0.002 | |  |
| Financial-Based Scarcity | -0.50 | -1.03 | -1.47 | | 0.46 | 0.30 | |  |
| Threat Exposure | -0.55 | -0.50 | -2.72 | | 1.63 | 0.62 | |  |
| **Financial-Based Scarcity * Threat Exposure** | 0.21 | 0.67 | -0.42 | | 0.84 | 0.50 | |  |
|  |  |  |  | |  |  | |  |
| *Thought Disturbance CBCL* | *F*(4, 127) = 0.66, *p* = 0.62, adjusted R^2^ = -0.01 | | | | | |  |  |
| Age | -0.09 | -1.55 | -0.20 | | 0.02 | 0.12 | |  |
| Financial-Based Scarcity | -0.00 | -0.03 | -0.27 | | 0.26 | 0.98 | |  |
| Threat Exposure | 0.01 | 0.03 | -0.59 | | 0.61 | 0.98 | |  |
| **Financial-Based Scarcity * Threat Exposure** | 0.01 | 0.09 | -0.17 | | 0.18 | 0.93 | |  |
| Note: CBCL = Child Behavior Checklist. Bold text indicates independent variable of interest. LL = lower limit, UL = upper limit. Because of power limitations in the split samples, the other indicator of environmental scarcity was not included as a covariate. | | | | | | | |  |


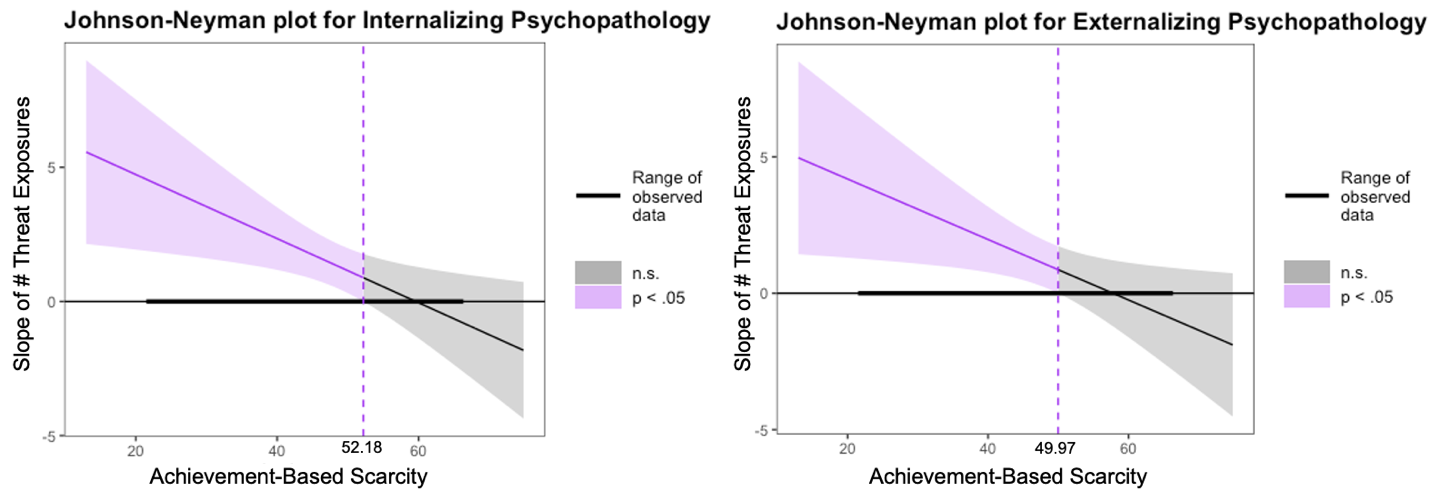
**Supplemental Figure 1**. Johnson-Neyman region of significance figures for internalizing and externalizing psychopathology. Purple dotted line indicates at what level of the moderator (i.e., scarcity indicator) the regression slope (i.e., threat exposure on psychopathology) is significant.

**Supplemental Figure 2**. Interactions between threat exposure and financial-based scarcity on internalizing, externalizing, and thought CBCL raw scores in each biological sex. Females shown in panels A-C (internalizing, externalizing, thought). Males shown in panels D-F (internalizing, externalizing, thought). Adjusted R^2^ and *p*-value represent those for the overall model. For visualization purposes only, data are split into one standard deviation above (solid dark line) and below (light dotted line) the mean (dashed line). CBCL = Child Behavior Checklist, SD = Standard Deviation.
